# Association between Hospital-acquired Disability and Clinical Outcomes in Older Patients Who Underwent Cardiac Surgical

**DOI:** 10.1298/ptr.E10263

**Published:** 2023-11-09

**Authors:** Hirokazu SUGIURA, Masahiro TAKAHASHI, Junichi SAKATA, Hiroki UCHIYAMA, Masanori NAKAMURA

**Affiliations:** ^1^Department of Rehabilitation, Sapporo City General Hospital, Japan; ^2^Department of Cardiovascular Surgery, Sapporo City General Hospital, Japan

**Keywords:** Hospital-acquired disability, Major adverse cardiac and cerebrovascular events, Cardiac surgery, Older adults

## Abstract

Objective: This study aimed to clarify the association between hospital-acquired disability (HAD) and prognosis in older patients who underwent cardiac surgery. Methods: This single-center, retrospective, observational study included 141 patients aged ≥65 years who underwent cardiac surgery at our hospital from November 2016 to August 2021. The primary endpoint of this study was the occurrence of major adverse cardiac and cerebrovascular events (MACCEs) within 2 years of hospital discharge. HAD was defined as a score of ≤5 on any one of the functional independence measure (FIM) subitems at discharge compared to preoperatively. Results: MACCE was observed in 16.3%, and the incidence of MACCE was significantly higher in the HAD group than that in the non-HAD group (12.1 vs. 34.5%, log-rank, p = 0.003). HAD was also significantly associated with the MACCE (hazard ratio [HD]: 2.575, 95% confidence interval [CI]: 1.001–9.655, p = 0.046). The incidence rate of HAD was 20.6%, with age (odds ratio [OR]: 1.260, 95% CI: 1.080–1.470, p = 0.004), preoperative short physical performance battery (SPPB) score (OR: 0.462, 95% CI: 0.301–0.708, p <0.001), and postoperative delirium (OR: 6.660, 95% CI: 1.480–30.000, p = 0.014) identified as significant factors. Conclusion: HAD is an independent predictor of MACCE in older patients who underwent cardiac surgery.

**T**he current population of individuals age ≥65 years in Japan exceeds 36.19 million, with the aging rate reaching 28.8%, which is estimated to increase to 30.0% by 2025 and 35.3% by 2040^1)^. Surgical indications have expanded to include older patients with more comorbidity, with the recent advances in surgical techniques and perioperative management, with >70,000 cardiac surgical procedures performed each year in Japan^2)^ and an increasing proportion of older patients^3)^. Preventing complications and reacquiring preoperative physical function and activities of daily living (ADLs) at an early stage is important in acute rehabilitation after cardiac surgery^4)^. However, many older patients have delayed progress in postoperative rehabilitation and are discharged from the hospital with a decline in ADLs compared with their preoperative ADLs.

Hospitalization-associated ADL decline is termed hospital-acquired disability (HAD)^5)^ and is known to occur in approximately 20%–40% of hospitalized older patients^6–8)^ and in 30% of older patients in a recent meta-analysis^9)^. Many previous studies have reported HAD as a strong prognostic factor in hospitalized older patients^10–12)^ and that HAD is an independent prognostic factor in patients with cardiac disease^13,14)^. Therefore, it may be a prognostic factor in older patients who underwent cardiac surgery. However, the prognostic impact of HAD development in older patients who underwent cardiac surgery is unclear.

Since multiple factors such as upper and lower limb muscle weakness, physical inactivity, balance function decline, and cognitive decline are associated with ADL decline in older patients^15–17)^, a single physical performance assessment alone may not be sufficient for evaluation. Monitoring HAD may provide important information to reconsider the care of older patients who underwent surgery if HAD is an independent prognostic factor.

We hypothesized that developing HAD in older cardiac surgical patients would impact prognosis. Therefore, this study aimed: 1) to clarify the association between HAD and cardiocerebrovascular prognosis in older patients who underwent cardiac surgery and 2) to explore factors associated with HAD development.

## Methods

### Participants

This single-center, retrospective, observational study included 201 consecutive patients aged ≥65 years who underwent cardiac surgery at our hospital from November 2016 to August 2021. Exclusion criteria were: 1) patients who were not independent in ADLs (any one of the subscores of the functional independence measure [FIM] was <5 points preoperatively), 2) emergency surgery, 3) patients who required intra-aortic balloon pumping or percutaneous cardiopulmonary support postoperatively, and 4) in-hospital death.

This study complied with the “Declaration of Helsinki” and the “Ethical Guidelines for Medical Research Involving Human Subjects” and was approved by the Sapporo City General Hospital Ethics Committee (approval number R03-061-915).

### Clinical outcomes

The primary endpoint of this study was the occurrence of major adverse cardiac and cerebrovascular events (MACCEs) within 2 years of hospital discharge. MACCE was defined as cardiovascular death and hospitalization for heart failure, myocardial infarction, angina pectoris, or peripheral arterial occlusive disease requiring revascularization, arrhythmia requiring catheter ablation or pacemaker implantation, cerebral infarction, or cerebral hemorrhage, arrhythmia, cerebral infarction, or cerebral hemorrhage requiring catheter ablation or pacemaker implantation. The primary endpoint was the presence of events and their duration during the 2 years after hospital discharge.

### ADL assessment and HAD definition

ADL performance was assessed preoperatively and at discharge by physical therapists using FIM^18,19)^. The FIM consists of 13 motor items, including eating, grooming, bathing, dressing the upper body, dressing the lower body, toileting, bladder management, bowel management, transfer to bed/chair/wheelchair, transfer to toilet, transfer to tub/shower, walking or wheelchair propulsion, and climbing stairs; and five cognitive items, including comprehension, expression, social interaction, problem-solving, and memory. The ADL evaluation method is a 7-point scale from 1 (full assistance) to 7 (complete independence), depending on the amount of assistance. A meta-analysis has confirmed its high reliability^20)^.

HAD was defined as a score of ≤5 (requiring monitoring and preparation by a caregiver) on any one of the FIM subitems at discharge compared to preoperatively^21)^.

### Preoperative data collection

Data collection included age; sex; body mass index; comorbidities; blood biochemical tests, such as C-reactive protein, serum albumin (Alb), serum hemoglobin (Hb), serum creatinine (Cre), and estimated glomerular filtration rate (eGFR); left ventricular ejection fraction by transthoracic echocardiography; percent predicted vital capacity (%VC); and forced expiratory volume in one second percent (FEV1.0%) by respiratory function tests.

### Assessment of preoperative physical function

A physical therapist preoperatively evaluated the short physical performance battery (SPPB)^22)^ and gait speed. The SPPB consists of three tests: balance, a 4-meter walk, and five times standing on a chair. The total score is the SPPB score (0–12). SPPB is the most recommended index in terms of reliability, validity, and feasibility among various physical function assessment indices for the older^23)^. Walking speed was measured twice using a 4-meter walking course to measure the walking time at a comfortable walking speed, and the faster value was adopted.

### Postoperative data collection

The following postoperative data were collected: type of surgery, operative time, anesthesia time, use of cardiopulmonary bypass, cardiopulmonary bypass duration, aortic cross-clamp time, blood loss volume, total fluid balance, and postoperative complications, including delirium, stroke, heart failure, arrhythmia, long-term ventilator management, and wound trouble. Heart failure was defined as postoperative low cardiac output or pulmonary congestion requiring cardiac insufficiency treatment such as inotropic drugs, arrhythmia was the postoperative appearance of new arrhythmia requiring defibrillation or pacemaker implantation, and long-term mechanical ventilation was postoperative ventilator management with intubation for ≥24 h. Wound problems were cases in which additional treatment, such as antibiotic therapy or debridement, was required due to wound infection.

The postoperative rehabilitation progress was examined in terms of the number of days patients started walking from the date of surgery, the number of days they walked 100 m independently, the number of days they stayed in the hospital postoperatively, and the rate of those discharged home. The 100-m walking independence was defined as the ability to continuously walk for ≥100 m in the hospital ward without supervision by a physical therapist, which is regardless of walking speed or the use of braces or self-help devices^24)^.

### Progression of postoperative rehabilitation

All patients underwent rehabilitation by physiotherapist intervention starting the day postoperatively. The postoperative protocol was based on The Japanese Circulation Society (JCS) guidelines for rehabilitation in patients with cardiovascular diseases^4)^. Thereafter, exercise therapy, such as aerobic exercise using a bicycle ergometer and treadmill and resistance training using weights and body weight, was performed. Rehabilitation was conducted 5–6 times a week, with 20–60 min of intervention per day, until the day before discharge.

### Statistical analysis

Continuous variables are presented as mean ± standard deviation (SD) for normal distributions and median (interquartile range) for non-normal distributions, and categorical variables are presented as numbers (%). The Shapiro–Wilk test was used to determine normality. Subjects were divided into two groups, the HAD and non-HAD groups, following the study definition. First, the unpaired t-test, Mann–Whitney’s U test, and chi-square test compared patient background factors between the two groups. Next, multivariate logistic regression analysis using the stepwise method was performed with HAD onset as the dependent variable. The Kaplan–Meier method was used to determine the incidence of MACCE within 2 years of hospital discharge, and the log-rank test compared the groups. Multivariate Cox proportional hazards analysis was performed with MACCE occurrence within 2 years of hospital discharge as the dependent variable and age, gender, and factors with p-values of <0.05 in the univariate analysis as independent variables. The multivariate analysis determined the correlation coefficients between independent variables to avoid multicollinearity, and absolute values were checked to ensure that they were not >0.8. As a subanalysis, the FIM subcategories that showed decline at discharge were classified into six FIM subcategories, and Cox proportional hazards analysis was performed with MACCE occurrence as the dependent variable and FIM subcategories as the independent variable. EZR version 1.55^25)^ was used for all statistical analyses, with a significance level of 5%.

## Results

### Study population and incidence of HAD

The analysis included 141 of the 201 patients in the study after excluding patients who were not independent in ADL preoperatively (n = 39), with emergency surgery (n = 16), patients who required intra-aortic balloon pumping or percutaneous cardiopulmonary support postoperatively (n = 2), and in-hospital death (n = 3).

HAD occurred in 29 of 141 (20.6%) patients. The FIM subscores that were <5 at discharge included eating (2 cases), grooming (3 cases), bathing (13 cases), dressing the upper body (6 cases), dressing the lower body (5 cases), toileting (4 cases), transfer to bed/chair/wheelchair (5 cases), transfer to the toilet (5 cases), transfer to tub/shower (9 cases), walking or wheelchair propulsion (6 cases), climbing stairs (20 cases), bladder management (3 cases), bowel management (4 cases), comprehension (2 cases), expression (3 cases), social interaction (4 cases), problem-solving (5 cases), and memory (3 cases).

Table 1 shows the preoperative patient background factors. The HAD group had significantly higher age and diabetes mellitus comorbidity and significantly lower Alb and Hb than the non-HAD group. The HAD group had lower preoperative SPPB scores and gait speed than the non-HAD group.

**Table 1.** Preoperative characteristics

	Overall (n = 141)	Non-HAD group (n = 112)	HAD group (n = 29)	p-value
Age, years	73 (69, 78)	72.5 (69, 77)	77 (71, 82)	0.004
Sex, female, n (%)	47 (33.3)	35 (31.2)	12 (41.4)	0.377
Body mass index, kg/m^2^	23.3 ± 3.1	23.3 ± 3.2	23.3 ± 2.9	0.904
LVEF, %	67 (57, 72)	66 (55, 71)	72 (67, 75)	0.052
%VC, %	92.8 ± 14.8	93.0 ± 14.7	92.1 ± 15.8	0.776
FEV1.0%, %	75.5 ± 8.1	75.4 ± 8.2	75.9 ± 7.5	0.752
Comorbidity				
Hypertension, n (%)	104 (73.8)	83 (74.1)	21 (72.4)	0.817
Dyslipidemia, n (%)	66 (46.8)	52 (46.4)	14 (48.3)	1.000
Diabetes mellitus, n (%)	87 (61.7)	64 (57.1)	23 (79.3)	0.033
Chronic kidney disease, n (%)	91 (64.5)	71 (63.4)	20 (69.0)	0.666
Chronic heart failure, n (%)	29 (20.6)	25 (22.3)	4 (13.8)	0.441
Chronic obstructive pulmonary disease, n (%)	10 (7.1)	10 (8.9)	0 (0)	0.123
Cerebrovascular disease, n (%)	30 (21.3)	24 (21.4)	6 (20.7)	1.000
C-reactive protein, mg/dL	0.1 (0.1, 0.3)	0.1 (0.1, 0.3)	0.1 (0.1, 0.7)	0.288
Albumin, g/dL	4.0 (3.7, 4.2)	4.1 (3.8, 4.3)	3.8 (3.5, 4.1)	0.002
Hemoglobin, g/dL	12.4 ± 2.0	12.6 ± 1.9	11.7 ± 2.1	0.021
Creatinine, mg/dL	1.0 (0.8, 1.4)	0.9 (0.8 ,1.3)	1.0 (0.7, 2.9)	0.512
eGFR, mL/min/1.73m^2^	53.3 (32.0, 64.9)	53.5 (40.2, 65.7)	42.2 (17.5, 63.0)	0.251
Preoperative SPPB score, points	11 (10, 12)	12 (11, 12)	9 (8, 11)	<0.001
Preoperative gait speed, m/s	0.93 ± 0.23	0.96 ± 0.22	0.75 ± 0.21	<0.001
Prepoerative FIM score, points	126 (125, 126)	126 (125, 126)	126 (124, 126)	0.662

Values are presented as mean ± standard deviation or median (interquartile range) or n (%).

HAD, hospital-acquired disability; LVEF, left ventricular ejection fraction; %VC, percent predicted vital capacity; FEV1.0%, forced expiratory volume in one second percent; eGFR, estimated glomerular filtration ratio; SPPB, short physical performance battery; FIM, functional independence measure

Table 2 shows the postoperative patient background factors. The HAD group had significantly longer cardiopulmonary bypass time and significantly higher blood loss volume, significantly higher rates of postoperative delirium and stroke complications, significantly longer postoperative time to 100-m walking independence and postoperative hospital stay, and significantly lower home discharge rates compared to the non-HAD group.

**Table 2.** Postoperative characteristics

	Overall (n = 141)	Non-HAD group (n = 112)	HAD group (n = 29)	p-value
Type of surgery, n (%)				0.130
CABG	49 (34.8)	43 (38.4)	6 (20.8)	
Valve surgery	36 (25.5)	27 (24.1)	9 (31.0)	
Combined valve surgery	24 (17.0)	15 (13.4)	9 (31.0)	
Combined CABG and valve surgery	9 (6.4)	8 (7.1)	1 (3.4)	
Other	23 (16.3)	19 (17.0)	4 (13.8)	
Operation time, min	399 (332, 497)	398 (324, 501)	421 (366, 493)	0.485
Anesthesia time, min	489 (417, 585)	475 (416, 595)	508 (441, 578)	0.537
Use of cardiopulmonary bypass, n (%)	89 (63.1)	66 (58.9)	23 (79.3)	0.052
Cardiopulmonary bypass time, min	166 (0, 241)	149 (0,236)	218 (130, 262)	0.040
Aortic cross-clamp time, min	104 (0, 151)	93 (0,150)	132 (89, 154)	0.060
Blood loss volume, mL	2715 (1,770, 3,865)	2663 (1644, 3585)	3450 (2620, 4620)	0.009
Total fluid balance, mL	2970 (2,207, 4,193)	2970 (2096, 4165)	3048 (2617, 4830)	0.298
Postoperative complication, n (%)				
Delirium	32 (22.7)	16 (14.3)	16 (55.2)	<0.001
Stroke	2 (1.4)	0 (0)	2 (6.9)	0.041
Heart failure	12 (8.5)	8 (7.1)	4 (13.8)	0.268
Arrhythmia	30 (21.3)	22 (19.6)	8 (27.6)	0.445
Long-term mechanical ventilation	18 (12.8)	11 (9.8)	7 (24.1)	0.058
Wound trouble	5 (3.6)	3 (2.7)	2 (6.9)	0.273
Initiation of walking, days	1 (1, 1)	1 (1, 1)	1 (1, 2)	0.088
100 m independent walking, days	5 (3, 6)	5 (3, 5)	6 (4, 9)	<0.001
Length of hospital stay after surgery, days	19 (16, 23)	18 (16, 23)	22 (18, 33)	0.010
Discharge to home, n (%)	104 (73.8)	90 (80.4)	14 (48.3)	0.002
Postoerative FIM score, points	125 (124, 126)	126 (125, 126)	118 (104, 122)	<0.001

Values are presented as median (interquartile range) or n (%).

HAD, hospital-acquired disability; CABG, coronary arterial bypass graft; FIM, functional independence measure

Table 3 shows the results of multivariate logistic regression analysis using the stepwise method with HAD onset as the dependent variable. Independent factors associated with HAD development were age (odds ratio [OR]: 1.260, 95% confidence interval [CI]: 1.080–1.470, p = 0.004), preoperative SPPB score (OR: 0.462, 95% CI: 0.301–0.708, p <0.001), and postoperative delirium (OR: 6.660, 95% CI: 1.480–30.000, p = 0.014).

**Table 3.** Independent risk factors for the patients with HAD

	OR	95% CI		p-value
Age (per 1 year increase)	1.260	1.080	1.470	0.004
Preoperative SPPB score (per 1 point increase)	0.462	0.301	0.708	<0.001
Delirium	6.660	1.480	30.000	0.014

Hosmer and Lemeshow goodness of fit test: χ^2^ value; 3.617, p = 0.890.

HAD, hospital-acquired disability; OR, odds ratio; CI, confidence interval; SPPB, short physical performance battery

### Association between HAD and the primary outcome

The median follow-up was 730 days (interquartile range: 455–730 days), and the follow-up rate was 95.7%. Further, 23 (16.3%) MACCEs occurred during the follow-up period, including heart failure (10 cases), angina (4 cases), arrhythmia (4 cases), peripheral artery occlusive disease (3 cases), and stroke (2 cases).

**Fig. 1.**
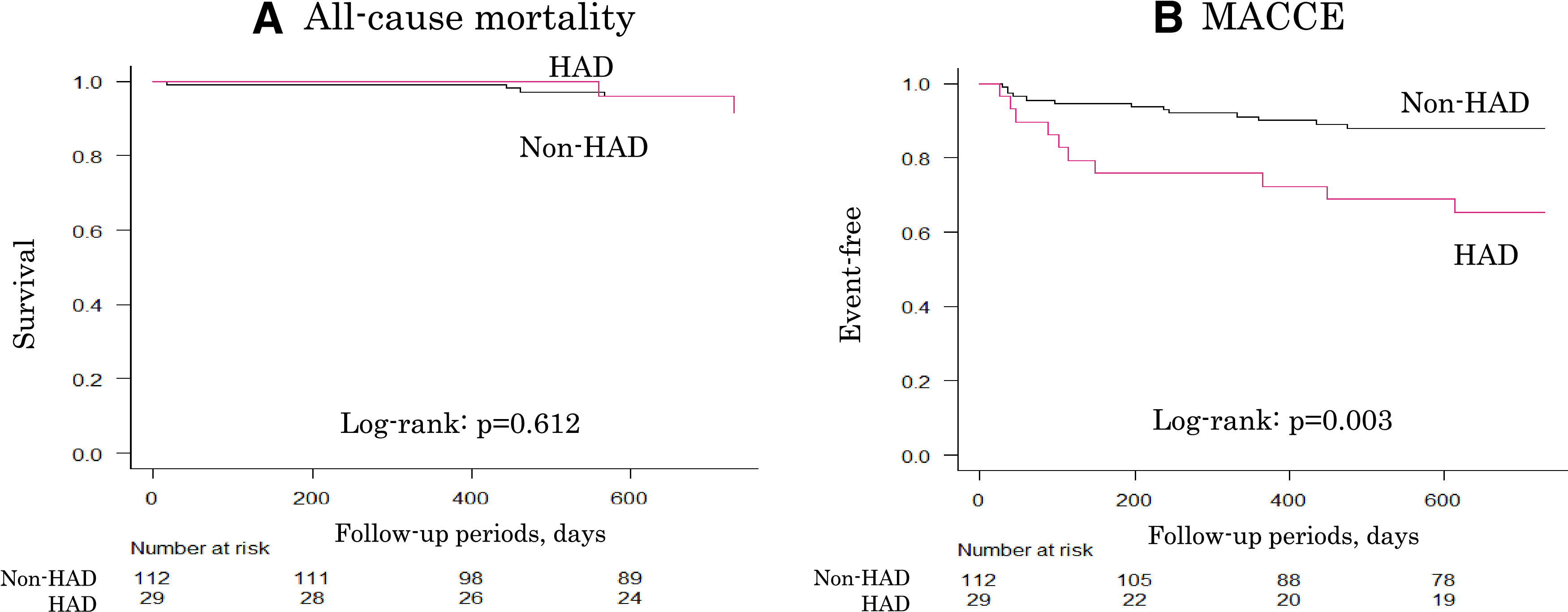
Kaplan–Meier curve for (A) all-cause mortality and (B) MACCE

MACCE, major adverse cardiac and cerebrovascular event; HAD, hospital-acquired disability

Figure 1 shows Kaplan–Meier curves. All-cause mortality demonstrated no significant difference (p = 0.612). Conversely, the incidence of MACCE was significantly higher in the HAD group than that in the non-HAD group (12.1% vs. 34.5%, log-rank, p = 0.003). Table 4 shows the univariate and multivariate Cox proportional hazards analyses. Multivariate Cox proportional hazards analysis revealed HAD as an independent poor prognostic factor using age and sex as adjusted variables and preoperative SPPB score, preoperative gait speed, postoperative hospital stay, and presence of HAD as independent variables ((hazard ratio [HR]: 2.575, 95% CI: 1.001–9.655, p = 0.046).

**Table 4.** Independent risk factors for MACCE after discharge

	Univariate analysis				Multivariate analysis			
	HR	95% CI		p-value	HR	95% CI		p-value
Age (per 1 year increase)	1.021	0.946	1.102	0.594	1.059	0.954	1.175	0.281
Male	0.926	0.393	2.184	0.860	1.294	0.432	3.877	0.645
Body mass index (per 1 kg/m^2^ increase)	0.891	0.776	1.023	0.101				
LVEF (per 1% increase)	0.985	0.951	1.019	0.371				
Hypertension	0.606	0.257	1.431	0.253				
Dyslipidemia	0.997	0.440	2.260	0.994				
Diabetes mellitus	1.423	0.585	3.461	0.436				
Chronic kidney disease	0.823	0.356	1.902	0.649				
Chronic heart failure	1.062	0.394	2.860	0.906				
Chronic obstructive pulmonary disease	0.556	0.075	4.125	0.566				
Cerebrovascular disease	0.788	0.268	2.318	0.666				
Albumin (per 1 g/dL increase)	0.567	0.234	1.356	0.202				
Hemoglobin (per 1 g/dL increase)	0.833	0.672	1.031	0.093				
Preoperative SPPB score (per 1 point increase)	0.771	0.635	0.937	0.009	0.933	0.589	1.477	0.766
Preoperative gait speed (per 1 m/s increase)	0.008	0.009	0.799	0.031	0.403	0.012	13.370	0.611
Type of surgery								
Combined CABG and valve surgery	1.000	Reference						
CABG	1.380	0.173	11.040	0.762				
Valve surgery	0.936	0.105	8.376	0.953				
Combined valve surgery	2.694	0.331	21.900	0.354				
Other	1.194	0.124	11.490	0.878				
Operation time (per 1 min increase)	0.998	0.998	1.003	0.846				
Cardiopulmonary bypass time (per 1 min increase)	1.001	0.998	1.004	0.520				
Blood loss volume (per 1 mL increase)	1.000	1.000	1.000	0.124				
Postoperative complication								
Delirium	1.820	0.772	4.294	0.171				
Stroke	0.001	0.001	26.098	0.997				
Postoperative day when ambulation independence was achieved (per 1 day increase)	1.020	0.909	1.144	0.735				
Length of hospital stay after surgery (per 1 day increase)	1.060	1.021	1.101	0.002	1.015	0.957	1.076	0.629
Hospital-acquired disability	3.220	1.411	7.347	0.005	2.575	1.001	9.655	0.046

MACCE, major adverse cardiac and cerebrovascular event; HR, hazard ratio; CI, confidence interval; LVEF, left ventricular ejection fraction; SPPB, short physical performance battery, CABG, coronary arterial bypass graft

As a subanalysis, we classified FIM subscores that showed a decline at discharge into six FIM subcategories and examined the association with MACCE. Table 5 shows the Cox proportional hazards analysis results with MACCE occurrence as the dependent variable and FIM subcategories as the independent variable. Self-care (HR: 3.384, 95% CI: 1.333–8.587, p = 0.010), mobility (HR: 2.112, 95% CI: 1.078–3.270, p = 0.046), communication (HR: 11.620, 95% CI: 3.843–35.150, p <0.001), and social cognition (HR: 6.667, 95% CI: 2.255–19.700, p <0.001) were identified as factors associated with MACCE.

**Table 5.** FIM subcategory and its relationship to MACCE after discharge

	HR	95% CI		p-value
Self-care	3.384	1.333	8.587	0.010
Sphincter control	0.983	0.133	7.295	0.987
Transfers	1.687	0.501	5.678	0.398
Mobility	2.112	1.078	3.270	0.046
Communication	11.620	3.843	35.150	<0.001
Social cognition	6.667	2.255	19.700	<0.001

FIM, functional independence measure; MACCE, major adverse cardiac and cerebrovascular event; HR, hazard ratio; CI, confidence interval

## Discussion

This study mainly revealed that HAD occurred in 20.6% of older patients who underwent cardiac surgery; preoperative SPPB score and postoperative delirium at this age were associated factors in HAD development. Most importantly, HAD was an independent predictor of MACCE within 2 years after discharge. To our best knowledge, this is the first report to define the onset of HAD using the FIM, which allows detailed and multidimensional ADL assessment and examines the prognostic impact of the onset of HAD in older patients who underwent cardiac surgery.

A previous study defined the incidence of HAD similarly in the present study, and the incidence of HAD in patients who underwent cardiac surgery was reportedly 18.1%^21)^. The present study reported almost the same incidence of HAD as that in the previous study (20.6%), and the results were considered valid.

This study revealed age, preoperative SPPB score, and postoperative delirium as independent factors associated with HAD development in older patients who underwent cardiac surgery. A combination of factors, including the age-related decline in physical reserve, cognitive and mental function, and decreased activity in the hospital setting, are associated with HAD development^5)^. A meta-analysis on functional decline during hospitalization revealed older age as a predictor of functional decline^26)^, supporting previous studies. A previous study of community-dwelling older subjects reported that SPPB scores were strongly associated with the future decline in ADLs, even after adjustment for age, gender, and background disease^27)^. A previous study of patients who underwent cardiac surgery reported older age and preoperative SPPB scores as independent factors associated with HAD development^28)^, and study results supported the previous study^5)^. Decreased physical activity in the hospital caused HAD^5)^; thus, the complication of postoperative delirium reduced the physical activity during hospitalization, which was associated with HAD development.

The primary endpoint revealed a significantly higher incidence of MACCE in the HAD group compared with the non-HAD group at 2 years after hospital discharge, and multivariate Cox proportional hazards analysis revealed HAD as an independent predictor of MACCE occurrence. The incidence of HAD has been an independently associated factor for poor prognosis in previous studies of hospitalized patients with acute internal medicine diseases and previous studies of patients with heart failure in Japan although the definitions of HAD differ^10,12–14)^. The present study revealed that HAD incidence has worsened the prognosis of cardiovascular disease, thereby supporting previous studies.

A previous study of older patients who underwent cardiac surgery reported that patients whose physical function, as assessed by the SPPB, declined during hospitalization had decreased instrumental ADLs (IADLs) and motor function after discharge, causing more severe frailty^29)^. Reportedly, the progression of frailty in patients who underwent cardiac surgery is associated with poor prognosis^30,31)^, and hospital discharge with HAD postoperatively may have decreased physical activity and IADLs at home, causing the progression of frailty and the occurrence of MACCE.

In addition, FIM subcategories that showed a decline at discharge were classified into six FIM subcategories and examined for association with MACCEs. The results showed that self-care, mobility, communication, and social cognition were significantly associated with MACCE. Therefore, conducting a detailed and multidimensional ADL assessment is essential.

The results of this study suggest that the presence of HAD should be considered in predicting the prognosis of older patients who underwent cardiac surgery. Additionally, preventing the onset of HAD and more careful follow-up of patients with HAD, including disease management programs by a multidisciplinary team and continued exercise therapy in the community, including transfer hospitals, to prevent the occurrence of MACCE, were considered important. Recently, preoperative rehabilitation for patients who underwent cardiac surgery is effective in improving physical function^32)^. Further, preoperative physical dysfunction in patients who underwent cardiac surgery is an independent factor associated with developing postoperative delirium^33)^, and preoperative rehabilitation intervention, as well as postoperative rehabilitation, may help prevent HAD development.

In recent years, advances in surgical techniques and perioperative management have expanded the indications for surgery to include older patients and those with critical illnesses, and the length of stay in acute care hospitals has also been shortened^34)^. Hence, the number of patients with HAD is expected to increase, indicating the clinical significance of this study.

### Limitations

This study has several limitations. First, this single-center study had a small sample size. Second, we excluded patients who were not independent in ADLs preoperatively or who required intra-aortic balloon pumping or percutaneous cardiopulmonary support postoperatively, which may have affected the results. Furthermore, the content of the ADL exercise and the discharge instructions were not standardized because this was a retrospective study. The individualized ADL practice and discharge instruction was conducted at the discretion of each physical therapist, which may have also affected the results, although the postoperative protocol was based on the JCS guidelines for rehabilitation in patients with cardiovascular diseases^4)^. Therefore, the uniformity of intervention methods should be examined in prospective studies in the future.

## Conclusion

HAD is an independent predictor of MACCE in older patients who underwent cardiac surgery. This study may assist in decision-making regarding the enhancement of acute postoperative rehabilitation and the provision of postdischarge social support to prevent HAD development.

## Conflict of Interest

There is no conflict of interest to disclose.
